# Comparison of ELISA with electro-chemiluminescence technology for the qualitative and quantitative assessment of serological responses to vaccination

**DOI:** 10.1186/s12936-020-03225-5

**Published:** 2020-04-17

**Authors:** Jessica S. Bolton, Sidhartha Chaudhury, Sheetij Dutta, Scott Gregory, Emily Locke, Tony Pierson, Elke S. Bergmann-Leitner

**Affiliations:** 1 Immunology Core, Malaria Biologics Branch, WRAIR, 503 Robert Grant Ave, 3W58, Silver Spring, MD 20910 USA; 2 Biotechnology HPC Software Applications Institute, Telemedicine and Advanced Technology Research Center, U.S. Army Medical Research and Development Command, Fort Detrick, Silver Spring, MD 21702 USA; 3 Dept. Structural Vaccinology, Malaria Biologics Branch, WRAIR, Silver Spring, MD 20910 USA; 4 PATH/Malaria Vaccine Initiative, Washington, DC 20001 USA

**Keywords:** Serology, Vaccine, Antigen, Multiplex, Antigenic competition, ELISA, Electro-chemiluminescence

## Abstract

**Background:**

Profiling immune responses induced by either infection or vaccination can provide insight into identification of correlates of protection. Furthermore, profiling of serological responses can be used to identify biomarkers indicative of exposure to pathogens. Conducting such immune surveillance requires readout methods that are high-throughput, robust, and require small sample volumes. While the enzyme-linked immunosorbent assay (ELISA) is the classical readout method for assessing serological responses, the advent of multiplex assays has significantly increased the throughput and capacity for immunoprofiling. This report describes the development and assay performance (sensitivity, linearity of detection, requirement for multiple dilutions for each sample, intra- and inter-assay variability) of an electro-chemiluminescence (ECLIA)-based multiplex assay.

**Methods:**

The current study describes the development of a multiplex ECLIA-based assay and characterizes the sensitivity, linear range, and inter- and intra-assay variability of the ECLIA platform and its agreement with the traditional ELISA. Special emphasis was placed on potential antigenic competition when testing closely related antigens in the multiplex format.

**Results:**

Multiplexing of antigens in ECLIA provides significant practical benefits in terms of reducing sample volume requirements and experimental time. Beyond the practical advantages of multiplexing, the ECLIA provides superior assay performance when compared to the ELISA. Not only does ECLIA show good agreement with the ELISA assay, but the linear range of ECLIA is also sufficiently wide to permit single-dilution measurements of concentration without the need to do serial dilutions. The lack of antigenic competition allows the simultaneous testing of closely related antigens, such as plate antigens representing different alleles of the same protein, which can inform about cross-reactivities—or lack thereof—of serological responses.

**Conclusion:**

The advantages of the newly developed tool for assessing the antigen profiles of serological responses may ultimately lead to the identification of biomarkers associated with various disease stages and or protection against disease.

## Background

Serological measures have long been used as either correlates of protection induced by a wide range of licensed vaccines targeting pathogens such as yellow fever, tetanus, polio, hepatitis A and B, measles, pertussis, rubella (reviewed in [1]), or as markers of exposure to a variety of pathogens [2]. Testing sera from preclinical and clinical studies has also been used to determine the potency of vaccine formulations as well as their potential to induce cross-species or cross-serotype reactive antibodies. Enzyme-linked immunosorbent assay (ELISA) have been the standard readout method to answer these aforementioned questions. The advent of multiplex testing platforms, such as the electro-chemiluminescence immunoassays (ECLIA), and bead-based flow cytometric assays enables the simultaneous detection for different antibody specificities and significantly increases the throughput of testing. The nature of multiplex platforms is ideal for sample sparing, enabling more in-depth analyses compared to single-plex assays such as the ELISA. Depending on the serological assay platform, antigens are either simply coated onto assay plates as in the case of the ELISA or they require modifications such as biotinylation or chemical linkage to fluorescent beads. In the case of the ECLIA, antigens require biotinylation to complex with proprietary linkers that allow targeted binding to specific regions in the assay well. The ECLIA technology tested here allows up to ten antigens to be coated in a single assay well.

The ECLIA follows the same logistics as the ELISA: assay plates are coated with antigens, then non-specific binding is reduced by a blocking step to exhaust remaining antigen-binding sites in the well, and finally, samples are added to the assay wells. Antibody binding to the plate antigens is detected by adding a secondary antibody specific for the immunoglobulin heavy chain of the antibodies in the test sample. For use in the ECLIA, the polyclonal secondary antibody is coupled with a proprietary Sulfo-Tag as the reporter molecule. Lastly, the substrate for the Sulfo-Tag is added. Upon inserting the specialized ECLIA plates into the reader, an electric pulse initiates the substrate conversion, resulting in chemiluminescence. A high-resolution camera quantifies the ECLIA signal in the various sectors of the well and reports the luminescence signal in each well sector. One of the significant advantages of ECLIA is that the substrate is activated by the reader thus eliminating any variability as result of timing associated with the addition of the substrate to the wells and the plates, which can be an issue in the ELISA.

A wide range of reagents are available for both the ELISA and the ECLIA, and several kits are available for clinical indication [3]. The results from the two platforms are typically reported as titres (OD1 titre or endpoint titre for ELISA) or mean luminescence signal for ECLIA. Quantitative data can be generated if a standard curve using purified immunoglobulins of a known concentration is run in parallel with the test samples for both assay platforms.

The present study describes a newly established ECLIA-based readout for malarial antigens using a model system in which human and nonhuman primate sera reactive to the circumsporozoite protein (CSP), the lead antigen for malaria vaccine development, were used as test sera. The plate antigens were either the full-length CSP [4], or peptides representing the central CSP-repeat region or C-terminal end of the CSP. Plate antigens with significant epitope-overlap were chosen deliberately to address potential antigenic competition when simultaneously testing sera for reactivity with different epitopes. The performance of the new ECLIA-based readout was compared to that of a qualified, malaria-specific ELISA performed in an international serology reference center, since the ELISA is a commonly employed serological readout in malaria (due to the relatively basic requirement for hardware), and historical comparison to earlier results within our program spanning close to 20 years [5, 6]. The ELISA requires testing of several replicates of a serially-diluted sample to either determine the OD1 titre or endpoint titre. In the case of a quantitative ELISA, several sample dilutions need to be tested to ensure that the OD of the sample falls within the linear range of the standard curve.

The objective of this study was to identify the serological assay platform that has the highest sensitivity, specificity, and linear range. Furthermore, the current study sought to determine whether simultaneous testing of closely related antigens in the same well of the assay plate was subject to antigenic competition.

## Methods

### Antigens and test samples

The antigens used for this study were derived from the sequence of the circumsporozoite protein (CSP, strain 3D7), the main surface protein of the *Plasmodium falciparum* parasite. The PfCSP-FL protein is comprised of 26_Tyr_–127_Asp_ linked to 207_Pro_–383_Ser_ [4]; “Repeat” is a 32-mer peptide representing the central Repeat region (NANP_8_); C-term is a recombinant protein representing the C-terminal fragment (AA 207-383); Pf16 is an epitope within the C-terminus that has been used as a functional marker when evaluating anti-CSP antibodies induced by vaccination [4, 7, 8]. To characterize the ECLIA platform and compare it to the classical ELISA, pre-existing CSP-immune nonhuman primate (NHP) samples (n = 30) [9] and a de-identified human CSP-immune serum pool were used. Commercial human pooled serum (Gemini Biosciences, Sacramento, CA) was used as negative (malaria-naïve) control serum. Two mouse monoclonal antibodies, one specific for the C-terminus of the CSP (clone 1E9, PATH/MVI), and one specific for the CSP-repeat region of the CSP (clone 1A6, PATH/MVI), were used as assay controls. The PfCSP-FL was biotinylated using the Lightning-Link Rapid Biotin Conjugation Kit (Expedeon, San Diego, CA) according to manufacturer’s instructions. The peptides were synthesized with a biotin-tag (Atlantic Peptides, Concord, NH).

### ELISA

The ELISA assay was performed in the Malaria Serology Laboratory (USMMRP, WRAIR Silver Spring, USA) employing full-length CSP, NANP peptide and C-terminal peptide (Pf16) as plate antigens as previously described [4, 10]. The coating concentrations of the plate antigens were 130 nM for CSP-FL, and 160 nM for the NANP repeat and Pf16 peptides. ELISA titres are listed as endpoint dilution at an optical density (OD) of 1.

### ECLIA

The described multiplex ECLIA methodology is based on the Mesoscale U-PLEX platform and 10-spot ECLIA plates (MSD, Gaithersburg, MD). An overview of the ECLIA platform regarding setup, assay logistics and data acquisition is given in Additional file 1: Fig. S1. Biotinylated proteins were diluted to desired concentrations using coating diluent (0.5% BSA, 1xPBS). All calculations were done based on molarity. 200 µl of each biotinylated protein (300 nM) was combined with 300 µl of a unique U-plex linker provided by the U-PLEX platform (MSD), vortexed, and then incubated at room temperature (RT) for 30 min. Post incubation, 200 µl of Stop Solution (MSD) was added to the biotinylated proteins and linker mix, vortexed, and incubated at RT for 30 min, resulting in a 10 × coating concentration. All U-PLEX-coupled protein solutions for the multiplexing were combined into one tube (600 µl each of the eight, U-PLEX-coupled protein solution). The U-PLEX-coupled protein solutions were brought up to 6 ml with Stop Solution, creating a 1× multiplex coating solution. Fifty µl of the 1× multiplex coating solution was added to each well of the U-PLEX 10-assay plates. Plates were sealed with sealing tape (Thermo Scientific, Waltham, MA) and incubated at RT for 1 h on a Titramax plate shaker (Heidolph, Schwabach, Germany), shaking at 700 rpm. Coated plates can be stored for up to seven days at 2–8 °C, based on manufacturer information.

After incubation, the plates were washed with a working solution of 1× MSD Wash Buffer (MSD) three times (150 µl/well). Sera were diluted to desired concentration with Diluent 2 (MSD) and added to each well (50 µl/well). The plates were sealed and incubated at RT for 1 h on a plate shaker (700 rpm). Plates were washed three times with 1× MSD Wash Buffer (150 µl/well). The detection antibody, SULFO-TAG goat anti-human antibody was diluted to 1 µg/ml in Diluent 3 (MSD) and added to the wells (50 µl/well). Plates were sealed and incubated at RT for 1 h on a plate shaker (700 rpm). After washing, 150 µl a working solution of 2× Read Buffer T (R92TC-3; MSD) was added to each well and the plates were read on the MESO QuickPlex SQ 120 (MSD), per manufacturer’s instructions.

### Statistical analysis

#### Calculating titres

For the ELISA assay, antibody titres for all four antigens were calculated using the linear extrapolation based on antibody dilutions closest to an OD of 1, to estimate the titre at OD = 1, as is standard practice in the WRAIR Malaria Serology Lab. For the ECLIA data, antibody titres were calculated using a 4-parameter logistic (4 pl) fit model [7]. The 4 pl model is to fit data from the entire titration curve, providing a more robust estimate of the titre at a particular signal intensity. ECLIA titres were calculated for a luminescence intensity of 10,000 Intensity Units (IU).

#### Bland–Altman analysis

The antibody titres for the ELISA and ECLIA assays were assessed for agreement using the Bland–Altman analysis for three antigens: CSP-FL protein, NANP CSP-repeat peptide, and C-term protein. The Bland–Altman analysis compares the *difference* in the ECLIA and ELISA titres (y-axis) with the *average* of the ECLIA and ELISA titres (x-axis). The Shapiro–Wilk test was applied to determine whether the differences between the two assays were normally distributed, using an alpha value of 0.05. If the differences were determined to be normally distributed, the standard deviation of the differences was used to determine the limits of agreement between which 95% of the differences would be expected to fall.

To determine whether there was a systematic trend in the difference between ECLIA and ELISA titres (ECLIA titre–ELISA titre) as a function of antibody concentration ((ECLIA titre + ELISA titre)/2), a linear regression analysis using *lm* function was carried out in the R statistical package. A linear fit was performed, then the 95% confidence interval of that linear fit estimated, and the statistical significance of whether the slope of that fit was non-zero determined. A non-zero slope would indicate a systematic trend in the discrepancy between the ECLIA and ELISA titres as a function of serum concentration.

#### Assessing linear range

The linear range of an instrument is the antibody concentration range where the read-out of a sample is proportional to the concentration. The linear range of the ECLIA assay was assessed in two ways. First, the correlation of the ECLIA luminescence intensity was measured at single-point dilutions with the antibody titres calculated using all the dilutions, across all samples. Second, to assess linearity directly, the change in signal intensity (Intensity, *I*) was calculated as a result of a change in antibody concentration (Concentration, *C*), or ΔIntensity/ΔConcentration, across the range of antibody concentrations and dilutions measured. Then the ΔI/ΔC curve was estimated by first plotting the ΔIntensity and ΔConcentration from consecutive data points in the correlation plot and then by applying a loess smoothing function using the *loess* function in R statistical package. The dilution and concentration span at which ΔI/ΔC ≈  1 is indicative of the linear range, while a ΔI/ΔC  ≈  0 indicates that the ECLIA instrument is either below its sensitivity limit (at low concentrations) or saturated (at high concentrations), and the readout is unresponsive to differences in antibody concentrations.

## Results

To establish a multiplex assay using an ECLIA platform, several parameters (i.e., antigen coating concentration, antigenic competition between closely related antigens, sample dilutions) were optimized and the performance of the assay determined in regards to specificity, linearity, and throughput. Four different, closely related antigens were tested to simulate potential field applications where either different epitopes of a given antigen or different alleles of the same antigen may be tested.

### Optimizing the ECLIA assay conditions

**Fig. 1:**
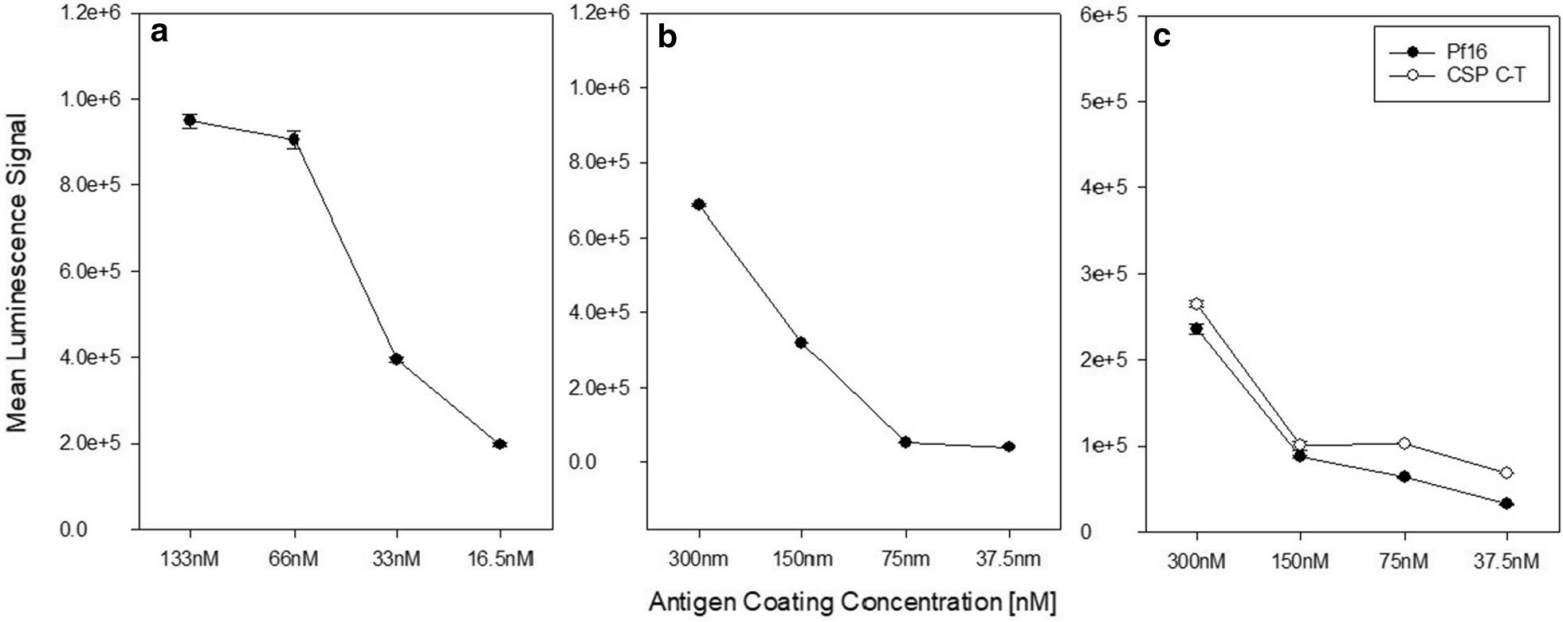
Optimization of coating concentrations for each antigen. Serial dilutions of CSP-FL **a**, peptide representing the CSP-repeat region **b**, and the two C-terminal peptides **c** were tested for reactivity with a human CSP-immune serum pool. The mean luminescence signal (MLS) of the malaria-naïve serum pool (negative control) did not exceed the background (i.e., wells incubated with secondary only (MLS < 1000 for all conditions)

The first step was to determine the optimal coating concentration for the ECLIA plates. Based on the manufacturer’s suggestions, the range of concentrations for the CSP-FL protein vs. CSP-derived peptides was based on the molecular weight (Fig. 1). The coating conditions for subsequent experiments were 66 nM for the CSP-FL protein and 300 nM for the peptides as these concentrations represented the upper end of the linear titration curves.

### Biotinylation does not alter the reactivity with CSP-immune antibodies

**Fig. 2:**
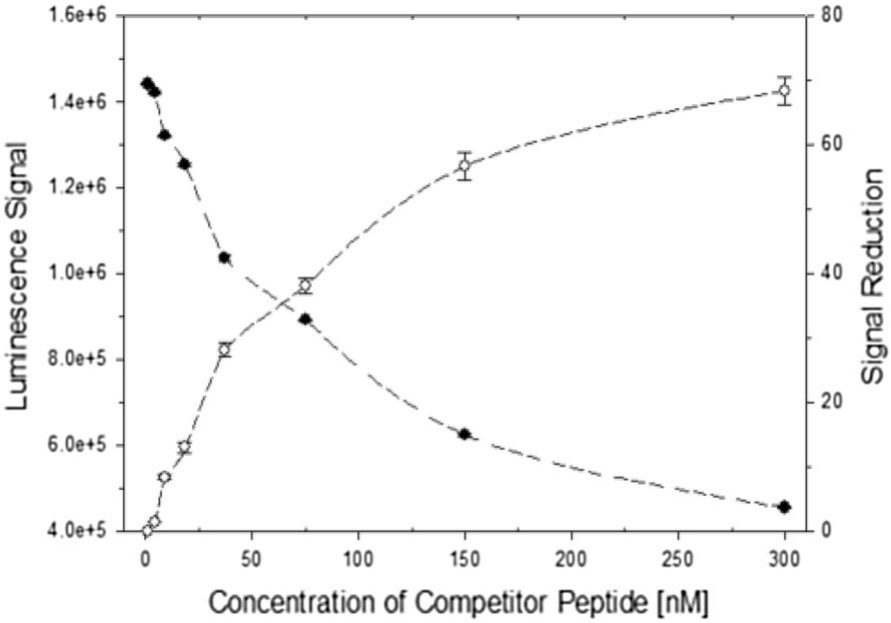
Biotinylation of peptide does not affect the reactivity with antibodies. Plates were coated with the biotinylated/U-PLEX linker-coupled competitor Pf16 peptide and unbiotinylated Pf16 peptide added at indicated concentrations to determine the ability to compete with the plate-bound antigen. Human CSP-immune serum pool was tested at 1:3000. Luminescence signal for the malaria-naïve, negative control (< 1200 MLS). Close circles represent the Luminescence signal, open circles the percentage signal reduction

A potential drawback of the ECLIA compared to the ELISA may be that antigens have to be biotinylated to enable coating of the assay plates. To demonstrate the impact of biotinylation on the reactivity of antibodies to the antigens, competition assays were set up to demonstrate specificity and epitope accessibility of the biotinylated, sector-specific and linker-coupled antigens (Fig. 2). ECLIA plates were coated with the biotinylated/-sector-specific linker-coupled Pf16 peptide using U-PLEX Linker 1. Malaria naïve pooled human serum (specificity control) and the CSP-immune serum pool were tested at a 1:3,000 dilution. Unlinked, non-biotinylated Pf16 peptide was used as competitor at 8 different concentrations (two-fold dilutions starting at 300 nM, which is the concentration of the linked plate antigen). Competing equal concentrations of plate-bound vs. soluble Pf16 peptide results in a roughly 70% competition. This could be due to different orientations and valencies when providing the peptide in a monomeric form vs. a format that may resemble multimers (due to the closer spatial arrangement of the peptide on the ECLIA well spot). In conclusion, biotinylation of the tested antigens does not alter the reactivity with CSP-immune antibodies.

### Equivalency of sector-specific U-PLEX linkers

**Fig. 3:**
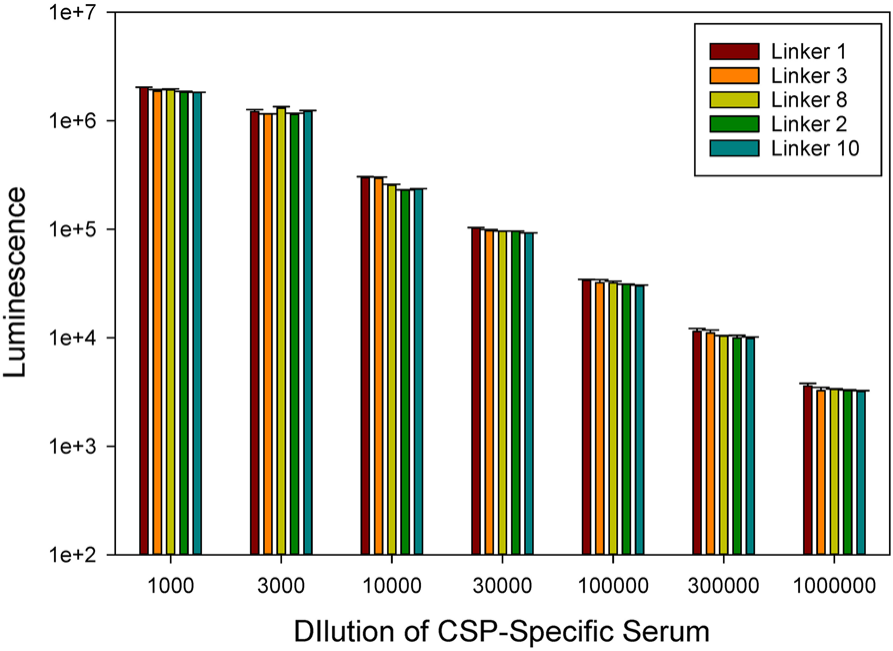
U-PLEX linker equivalency for PfCSP (3D7) C-term peptide. The peptide was linked with the five different, randomly selected linkers and then ECLIA plate wells coated in a singleplex format. CSP-immune pool was used at dilutions indicated on x-axis to detect potential differences in the equivalency of the linkers. The signal with the negative control serum (specificity control) did not exceed MLS < 1000. Data expressed as mean luminescence signal (MLS) (± SD) of two independent experiments).  % CV was less than 5% for all tests

The next step in optimizing the assay conditions for a quantitative multiplex assay that is able to test closely related antigens in parallel was to determine whether the various U-PLEX linkers were equivalent and did not introduce a bias in the analysis. Biotinylated protein aliquots were complexed with U-PLEX linkers 1, 2, 3, 8, 10, and plates were coated with the different U-PLEX-coupled antigen solutions. A wide range of dilutions (1:1,000- 1:1,000,000) of a human CSP-immune serum pool was tested to determine potential quantitative differences in the luminescence signal (Fig. 3). The results demonstrated that the U-PLEX linkers were equivalent and differences in signal strength only reflect differences in the fine specificity of test samples.

### Multiplexing of closely related antigens is not subject to antigenic competition

**Fig. 4:**
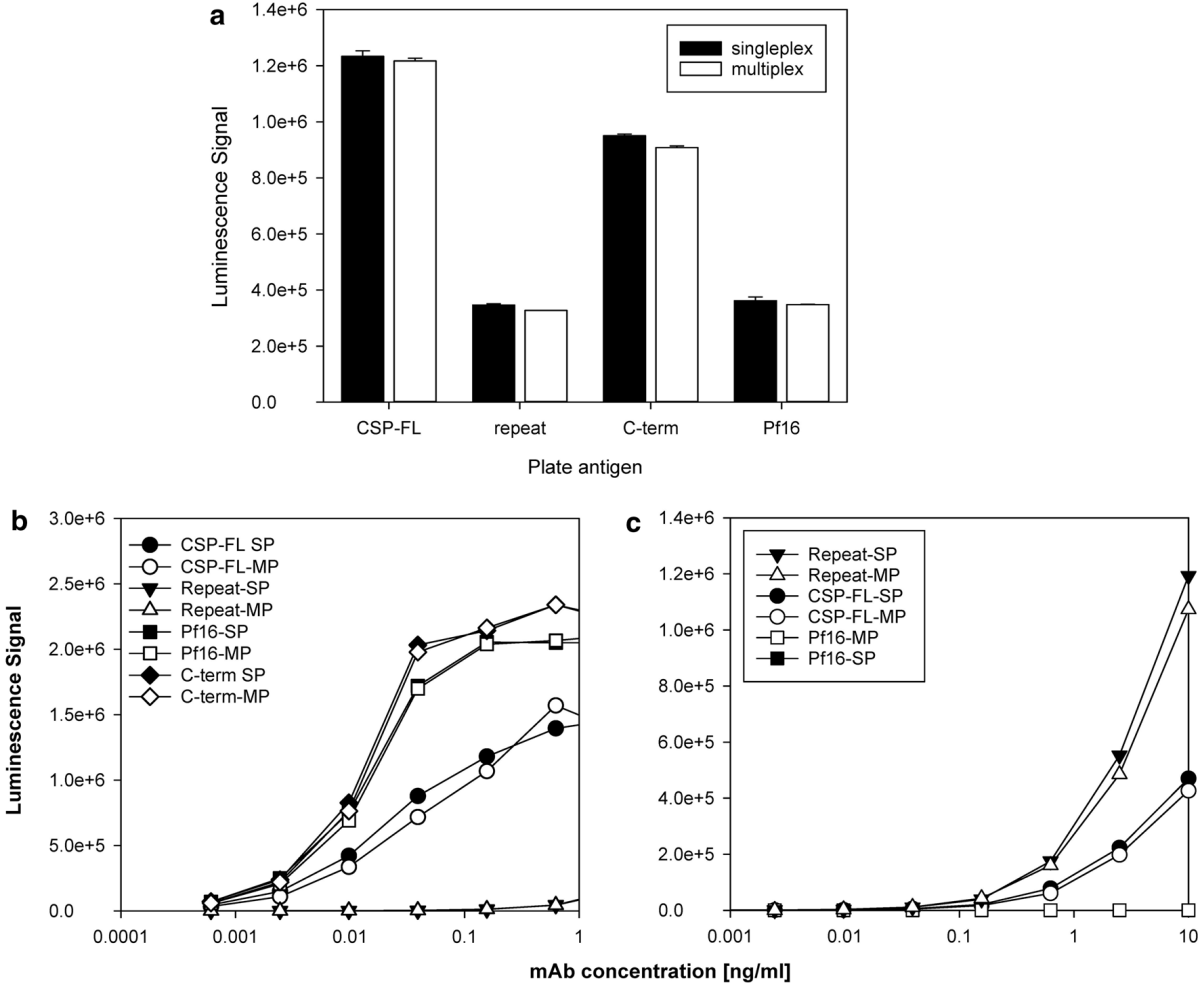
Testing of closely related antigens to identify antigenic competition. Human CSP-immune serum pool (1:5000 dilution) tested for reactivity against either singleplexed antigens (SP) or multiplexed antigens. **a** In addition, titrations of CSP C-terminus specific mouse mAb 1E9 **b** and CSP-repeat-specific mAb 1A6 **c** were tested reactivity against either singleplexed antigens (SP) or multiplexed antigens. Data are expressed as mean luminescence signal (MLS) of two independent experiments for each panel.  % CV was less than 5% for all tests. The luminescence signal with malaria-naïve serum (specificity control) did not exceed MLS < 1000

To enable multiplexing of closely related antigens, it was important to determine whether such antigens compete with each other for binding to antibodies in the sample (Fig. 4). The multiplexed ECLIA experiment tested the full length antigen (CSP-FL), its central repeat region (NANP), its C-terminal fragment protein (C-term) and the smaller C-terminal peptide Pf16. The optimal coating concentrations (66 nM for CSP-FL, 300 nM for the fragments/protein subunits) were applied to coating either wells with one antigen only (singleplex) or a cocktail consisting of all peptides (multiplex). CSP-immune serum and malaria-naïve human pooled serum (negative control) were used at 1:5000 dilution to determine whether multiplexing resulted in lower luminescence signal, which would indicate antigenic competition (Fig. 4a). To demonstrate specificity of the response, mouse monoclonal antibodies (mAbs), specific for the C-terminus or the CSP-repeat region, were tested against all plate antigens (Fig. 4b, c). Titrations of these mouse mAbs were performed to demonstrate that no antigenic competition occurs at any antibody concentration and to further establish specificity of the responses in the single- vs. multiplexed format. The C-terminus specific mAb 1E9 did not react with the CSP-repeat peptide (Fig. 4b) and the CSP-repeat -specific mAb 1A6 did not react with the C-terminal fragments of CSP (Fig. 4c). It is noteworthy that the titrations of the mAbs showed a different dose response curve; mAb 1E9 yielded a typical response curve with a linear portion and a saturation point for both the CSP-FL and the C-terminal fragments. In contrast, the titration of the CSP-repeat-specific mAb 1A6 did not reach saturation despite a wide range of concentrations. Both mAbs responded stronger with their respective fragment than with the CSP-FL. In summary, no antigenic competition was detected when using either CSP-immune human serum or mouse monoclonal antibodies as evidenced by comparable signal strength in the singleplex and the multiplex assay format.

### Inter-assay variability of CSP-based ECLIA readout method

**Fig. 5:**
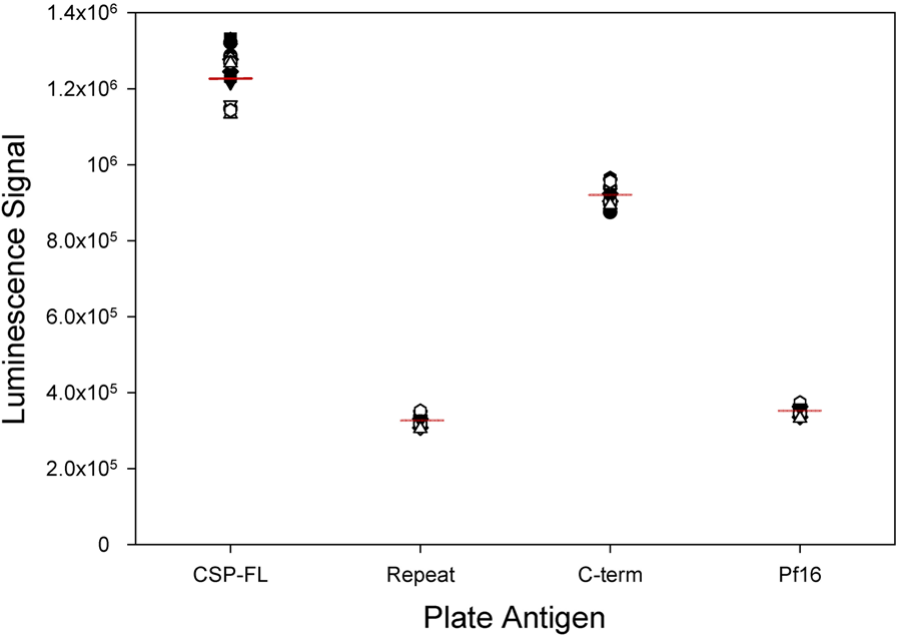
Inter-assay variability of the multiplex ECLIA-based serological testing platform. A CSP-immune serum pool was run at 1:5000 dilution in the course of eight months by two operators. Dot plot representing the mean luminescence signal (MLS) of n = 8 experiments (16 data points) for each plate antigen. Signal of a CSP-naive serum pool (run as negative control in each experiment) did not exceed MLS < 1000 for any antigen

The variability of the ELISA platform has been well documented [11]. The accepted  %CV in the Malaria Serology Laboratory (WRAIR) has been ≤ 15% for the plate antigens described in the current study. To complete the characterization of the ECLIA platform, human CSP-immune and control serum pools were repeatedly tested over the course of eight months and by two operators to determine the robustness of the data obtained with this assay platform (Fig. 5). Using CSP-immune human serum, there was a clear hierarchy in the reactivity to the different plate antigens: the highest reactivity was against the CSP-FL followed by the C-terminal fragment, the C-terminal protein (Pf16), and the CSP-repeat peptide. This may reflect the number of epitopes that are available for antibody binding or may indicate the need for some conformation. Overall, the minor variability (≤ 5.4% CV for CSP-FL, ≤ 4.8% CV for CSP-repeat, and ≤ 3.7% CV for both C-terminus antigens) in the results indicate that this assay was highly reproducible and significantly lower than the variability of the ELISA.

### Performance comparison of singleplex ELISA and multiplex ECLIA

**Fig. 6:**
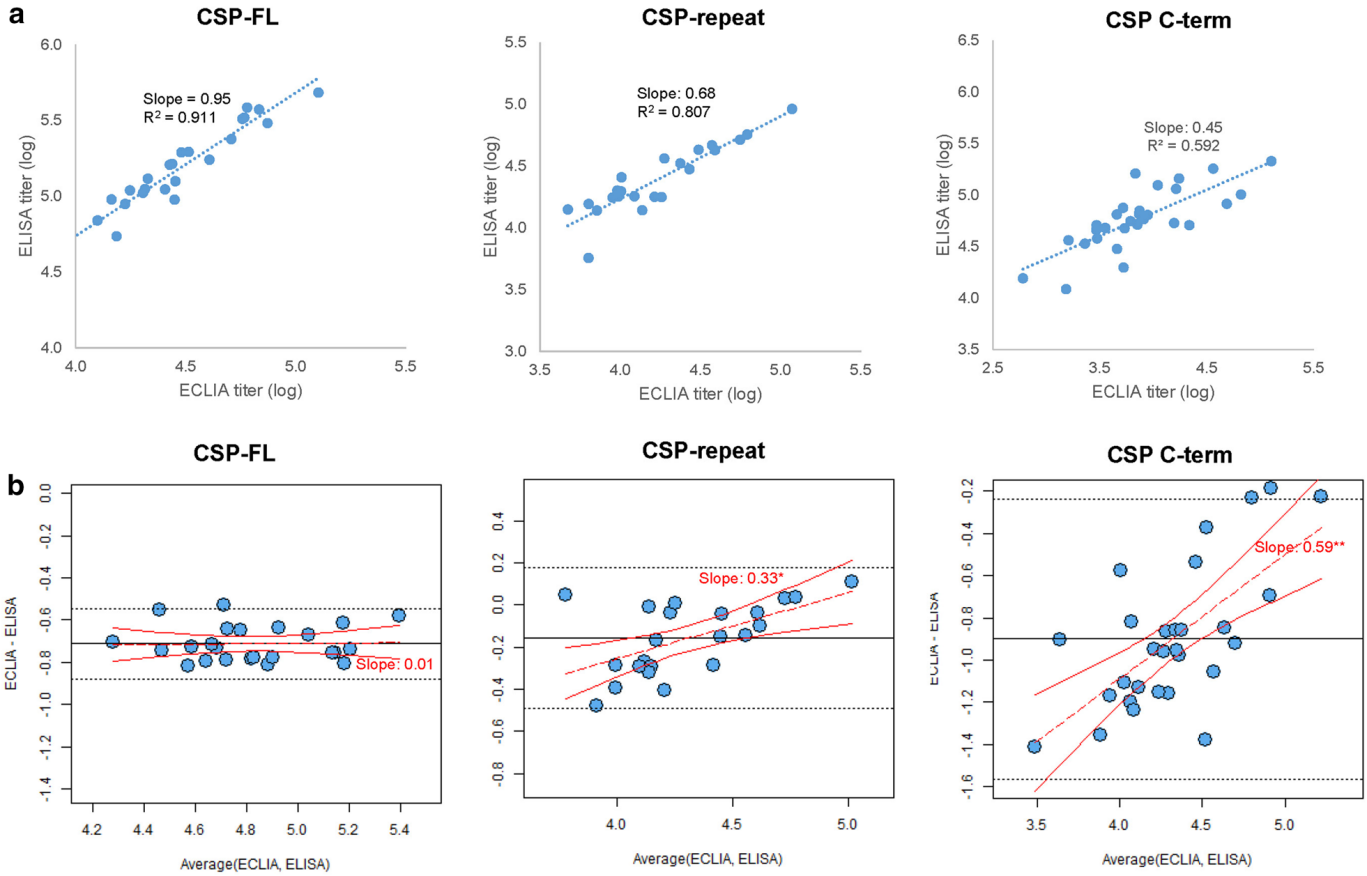
**a** Correlation plots of the ELISA and ECLIA titres for CSP-FL (left), CSP-repeat (NANP; center), and C-term (right) antigens from samples from 30 NHP animals immunized with a particle-based CSP vaccine. Slope and correlation coefficient are shown. **b** Bland–Altman plots comparing ELISA and ECLIA assays for the aforementioned antigens. The mean difference between the ELISA and ECLIA titres (black line) and the 95% confidence interval of the difference (dotted black line) are shown. The systematic trend in the difference in ELISA and ECLIA titres as a function of antibody concentration is represented by the slope (dashed red line), along with the 95% confidence interval of the slope (solid red line). The slope is labelled along with a corresponding p-value (*p < 0.01, **p < 0.001)

A sample set of 30 nonhuman primates immunized with a particle-based CSP vaccine [9] were tested in parallel by an established, qualified ELISA [8, 10] vs. the multiplex ECLIA assay. Samples were serially diluted (range of 1:50 to 1:6400 for ELISA and 1:500 to 1:64,000 for ECLIA) and simultaneously tested in both assays. For the ELISA data, antibody titres were calculated using linear extrapolation, as is common practice. For the ECLIA data, antibody titres were calculated using a 4-parameter logistic fit model [7], which better accommodates the wide dilution range used in this assay. A correlation analysis was carried out comparing the ELISA and ECLIA titres for three antigens: CSP-FL, CSP-repeat, and CSP C-term (Fig. 6a), followed by a Bland–Altman analysis to assess the level of agreement between the two assays (Fig. 6b).

For the CSP-FL antigen, there was a high level of agreement between the ELISA and ECLIA titres. The titres from the two assays had an R^2^ of 0.911, and the Bland–Altman analysis showed that the ECLIA titre was within 1.46-fold of the ELISA titre with 95% confidence. Furthermore, although there was an absolute bias in the ECLIA titres relative to the ELISA titres, which is to be expected as titres are a relative measure of concentration, there was no systematic trend in the discrepancy between ECLIA and ELISA titres across the concentration ranges measured here. For the CSP-repeat antigen, there was also good agreement between the two assays, with an R^2^ of 0.81, and ECLIA titres found to be within 2.1-fold of the ELISA titres with 95% confidence. However, unlike in the case of the CSP-FL antigen, there was a systematic trend of increasing difference between ECLIA and ELISA titres at lower antibody concentrations, indicated by a slope of 0.33 in the Bland–Altman plot (p < 0.01). This suggests that the ECLIA assay may be more sensitive than the ELISA assay at these low concentrations. For the C-term antigen, there was moderate agreement between the ELISA and ECLIA titres, with a R^2^ of 0.45, and ECLIA titres found to within 4.5-fold of the ELISA titres with 95% confidence. As for the CSP-repeat antigen, the Bland–Altman plot revealed a systematic trend of increasing difference between the ECLIA and ELISA titres at lower antibody concentrations (slope 0.59, p < 0.001), again suggesting that the ECLIA assay may be more sensitive at lower concentrations.

### Quantitative differences between ELISA and multiplex ECLIA in assay performance

One important question for high-throughput screening is whether sample testing needs to be done at multiple dilutions. Although time consuming and resource intensive, multiple dilutions are often necessary to ensure that all the samples are measured at least once by the instrument within its range of linearity–that is, the concentration range where the readout is linearly related to the concentration. Outside of this range, for example, below the sensitivity of the instrument or above the concentration where the signal is saturated, the readout no longer reliably reflects antibody concentrations. Therefore, the next step was to assess the linear range of the two assay platforms ECLIA and ELISA.

**Fig. 7:**
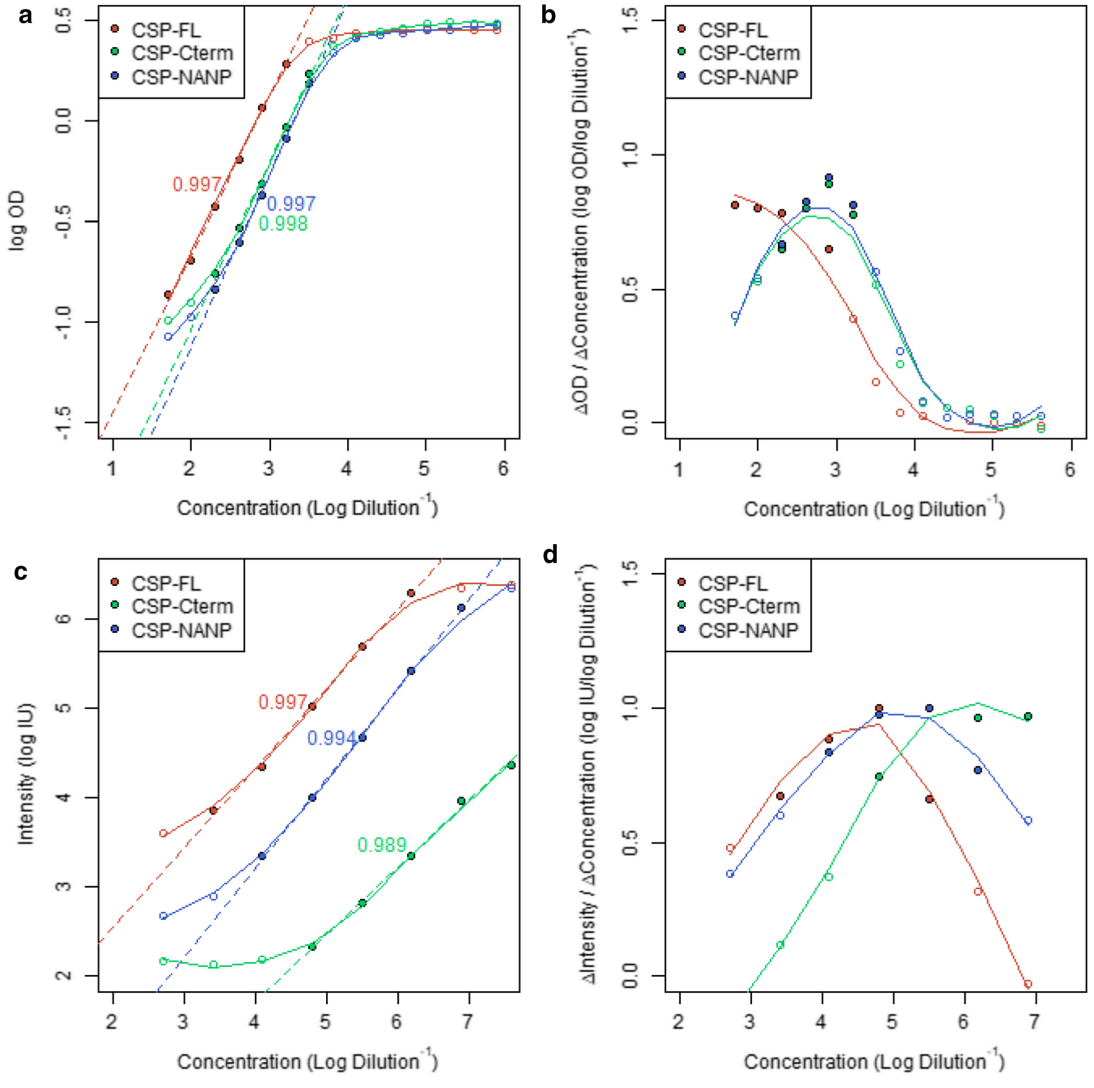
Linearity of ELISA vs ECLIA based assay for the assessment of CSP-specific antibodies. **a** ELISA readout for single sample (human CSP-immune pool) across fifteen twofold dilutions from 1:50 to 1:819,200, compared against the relative serum concentration, expressed as the log Dilution^−1^ for the CSP-FL, CSP C-term, and NANP antigens. Dilution points that were found to be in the linear range are marked (solid circles), with corresponding R^2^ values. **b** ΔOD/ΔC values calculated between adjacent dilutions against relative serum concentration for the ELISA. ΔOD/ΔC curve estimates are shown as well. **c** ECLIA assay readout for a single sample (human CSP-immune serum pool) across eight fivefold dilutions against CSP-FL, CSP C-term, and NANP against the relative serum concentration, expressed as the log Dilution^−1^. Five dilution points (solid circles) were found to be in the linear range for each antigen, with corresponding R^2^ values shown. **d** ΔI/ΔC values calculated between adjacent dilutions against relative serum concentration for the ECLIA. ΔI/ΔC curve estimates are shown as well

Towards that end, serially diluted human CSP-immune serum pool was tested across 15 twofold dilutions, from 1:50 to 1:819,200 (Fig. 7a) and the serum samples were measured against three antigens in the ELISA: CSP-FL, CSP C-term, and CSP-repeat (NANP). The readout showed linear behavior over a serum concentration range of approximately 64-fold (six twofold dilutions. The change in ELISA signal intensity was calculated as a function of a change in antibody concentration (ΔI/ΔC) for the fifteen dilutions to assess the degree of linearity (Fig. 7b). An estimated *Δ*I/*Δ*C near 1.0 would indicate perfect linearity, while a *Δ*I/*Δ*C of 0.0 would indicate either being below the sensitivity limit of the instrument (at low concentrations), or saturation of the instrument (at high concentrations). At dilutions of 1:50 to 1:400 for CSP-FL, and 1:200 to 1:3200 for C-term and CSP-repeat, the *Δ*I/*Δ*C was near 1.0, indicating some degree of linearity. This corresponds to a linear range of approximately tenfold concentrations.

To measure the linear range of ECLIA, serially diluted human CSP-immune serum pool was tested across eight fivefold dilutions, from 1:500 to 1:39,062,500 (Fig. 7c) and the serum samples were measured against three antigens: CSP-FL, CSP C-term, and CSP-repeat (NANP). The ECLIA assay readout showed linearity for a range of five fivefold dilutions for all three antigens, encompassing a 625-fold range in antibody concentrations. The signal intensity showed robust linearity with relation to concentration, achieving *Δ*I/*Δ*C greater than 0.0, and close to 1.0, across this wide range of concentrations for all three antigens (Fig. 7d). These findings not only demonstrate the wide linear range of the ECLIA assay platform, but also highlight its high sensitivity even at very low antibody concentrations. For CSP-FL- and CSP-repeat-specific antibodies, the highest dilution still exceeded the sensitivity limit of the instrument, while for CSP C-term, the three highest dilutions did appear to be below the sensitivity limit.

**Fig. 8:**
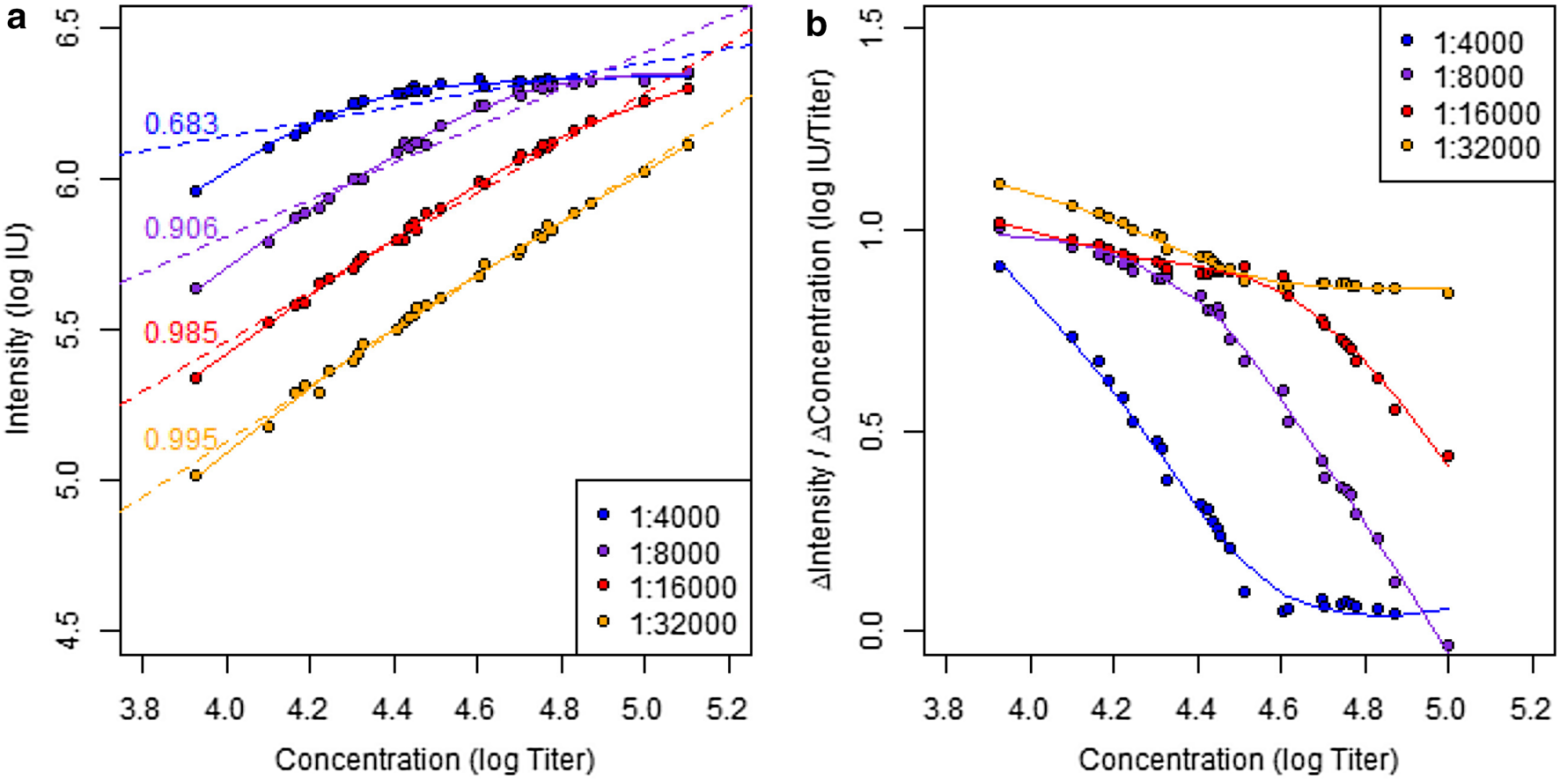
Assessing the single-dilution readouts for ECLIA assay. **a** ECLIA assay readout (Intensity, in Intensity Units (IU) at four single-point dilutions (1:4000, 1:8000, 1:16,000, and 1:32,000) against CSP-FL compared with ECLIA antibody titres calculated using all the dilutions of the 30 NHP serum samples. A linear model was fit to the data and the corresponding R^2^ values is shown. **b** ΔI/ΔC values calculated between adjacent antibody titres is shown for the four single-point dilutions against the antibody titres for each sample in the study

Given the wide linear range of ECLIA, the next step was to determine whether single-dilution measurements of the biological samples would be sufficient to accurately determine serum concentration across all the samples against the CSP-FL antigen. The biological samples tested here were from thirty CSP-immune nonhuman primates. Towards that end, the single dilution read-out was compared across four dilutions (1:4,000, 1:8000, 1:16,000, 1:32,000) with the titre calculated from the serial dilutions as the ‘gold standard’ (Fig. 8a). For the four single-point dilutions analysed, R^2^ values as high as 0.995 for the highest dilution were observed, over a serum antibody concentration range of approximately tenfold. The 1:4000 and 1:8000 dilutions did show evidence of saturation for samples with higher antibody concentrations. At the 1:16,000 and 1:32,000 dilutions, the relationship between signal intensity and concentration was highly linear, with *Δ*I/*Δ*C of close to 1.0 across most of the serum antibody concentration range (Fig. 8b). By contrast, 1:4000 and 1:8000 showed *Δ*I/*Δ*C approaching 0.0 for samples with higher antibody concentrations, indicating some degree of instrument saturation. These findings suggest that testing the thirty CSP-immune NHP serum samples at a single dilution (1:32,000) was sufficient to accurately determine the antibody titres by ECLIA.

## Discussion

The present study describes the features of a newly developed serological panel that is based on a multiplex ECLIA-assay platform. Comparing some of the features with the classic ELISA demonstrated the advantages of the ECLIA based assay for assays that establish the antigenic profile of humoral immune responses in either vaccinated individuals or residents of malaria-endemic areas (summarized in Table 1). Special emphasis was placed on determining whether closely related antigens could be tested simultaneously without impacting the quantification of such antibodies. To this end, a single malarial antigen, CSP, and its fragments were used as plate antigens. CSP is one of the leading malaria vaccine antigens [12]; the magnitude of antibody responses to either full length CSP [13] or its fragments has been identified as a potential biomarker of protection [14, 15]. While the role of CSP-repeat-specific antibodies has been well documented [8, 13, 15, 16], there are conflicting data on the role of C-terminus-specific antibodies [7, 14, 17] and their ability to contribute to protection against infection. The method described here enables high-throughput testing and permits profiling of large samples sets even when sample volumes are limited to determine the role of epitope specificity of CSP-specific antibodies.

**Table 1:** Summary of features characteristic to ELISA vs. ECLIA

Parameters	ELISA	ECLIA
Cost of hardware	Starting at $13,000	Starting at $54,000
Cost of assays	High (due to singleplexing)	Medium (high throughput and multiplexing)
Throughput	24 dilutions of samples per plate (Triplicates) → 6 samples/plate	80 samples/plate (singlicates);
	8 plates/day → 48 samples/day	8 plates/day → 640 samples
Operator skill	Basic	Basic
Training time	Weeks	Weeks
Hands-on time	1 h	2 h
Asssay length	5 h (plus ON plate coating)	6.5 h
Reporter	Enzymatic	Electro-chemiluminescence
Detection	Colorimetry	Luminescence
Results reported	OD1 titer	Mean Luminescence Signal
	Endpoint titer	Titer
	Concentration	Concentration
Chemistry	Absorption to protein binding plates	Biotinylated antigen binding to U-PLEX linkers
Assay format	Plate based	Plate based
Multiplexing	No	Yes
Number of analytes	1 analyte/well	1–10 analytes/well
Serial dilutions needed	Yes	No
Sample volume	> 5 µl sample for one antigen (in most instances)	< 0.1 µl sample for 10 antigens
Linear range	Narrow	Wide
Sensitivity	Low	High

The assay development report evaluates crucial parameters for a sensitive and reproducible assay: (1) the optimal coating concentrations for the CSP protein, as well as the derived peptides that represent important functional elements in efficacious immune responses induced by vaccination; (2) the equivalency of the U-plex linkers to ensure that no bias is introduced by assigning test antigens to linkers that are not capable of delivering the same signal strength; (3) the impact of biotinylation on immunoreactivity with specific antibodies. In this study, biotinylation did not notably change the interaction between antibodies and the antigens. However, it should not be assumed that this will be the case for all antigens. One cannot exclude the possibility that biotinylation may impact access to select epitopes by a subset of antibodies due to steric hindrance. The assay described here is intended for the characterization of polyclonal responses where steric hindrance would only have a negligible impact on the overall signal considering the wide range of epitope specificities and the high sensitivity of the assay platform. Other multiplex platforms such as bead-based flow cytometry also requires modification of the antigens by either biotinylation or chemical linkage. The latter assay platform has been used in some field studies for the profiling of antibody specificities and their contribution to clinical outcomes [18, 19]. Alternatively, protein arrays are available that allow an in-depth profiling of the antibody responses in vaccinees or residents of malaria endemic areas with special emphasis on polymorphic antigens where several alleles have been included into the array chips [20]. The ECLIA method described here could be considered complementary to protein microarrays. While microarrays typically contain hundreds of antigens printed onto chips and can be costly, they are instrumental in profiling serological responses and are invaluable in identifying biomarkers of protection [21]. Once specific markers of protection or disease have been identified, they could be applied to the ECLIA assay platform, thus streamlining the testing process and reducing the overall cost of assay performance and analysis.

## Conclusion

The present study demonstrates the superiority of the ECLIA based serological assay over the conventional ELISA. The two assays show strong quantitative agreement. However, because of the extremely wide linear range of the ECLIA, a simple single-point measurement is sufficient to determine antibody titres. By contrast, in the ELISA, a much narrower linear range means that multiple dilution points are necessary for each sample to be in the linear range of the instrument, and then serial dilutions are required to create a titration curve from which a titre can be calculated. Furthermore, the ECLIA can be multiplexed to measure responses to multiple antigens simultaneously from a single sample. Equally important, no antigenic competition could be detected when testing closely related antigens in the ECLIA. These characteristics make the ECLIA the preferred platform for serological immunoprofiling, which is crucial for the identification of biomarkers of exposure or correlates of immunity.

## Supplementary information

**Additional file 1: Figure S1.** Overview of ECLIA assay platform. (Panel A) Experimental steps for assay setup. Biotinylated antigens are coupled with proprietary U-PLEX linkers in separate tubes. Once coupling is complete, U-PLEX-coupled antigens are combined into a cocktail and the assay plates coated. Each U-PLEX linker can only bind to its respective spot (color coded in Figure); up to 10 antigens can be coated per well (top view plate well). (Panel B) Overview of all steps to complete the assay: antigen-coated wells are incubated with diluted serum or plasma. Antigen-specific antibodies will bind to the antigen and the binding visualized by adding a Sulfo-Tag-labeled secondary antibody and substrate. (Panel C) Data acquisition. Plate is inserted into reader which will deliver an electric pulse that activates the substrate. A high resolution camera measures the luminescence above each spot.

## Data Availability

The data and detailed protocol can be made available upon request from the corresponding author.

## Abbreviations

BSA: Bovine serum albumin
CSP: Circumsporozoite protein
CSL-FL: Full length CSP
CV: Coefficient of variation
ECLIA: Electro-chemiluminescent immune-assay
ELISA: Enzyme linked immunosorbent assay
IU: Intensity units
mAb: Monoclonal antibody
MLS: Mean luminescence signal
NHP: Nonhuman primate
OD: Optical density
ON: Overnight
PBS: Phosphate buffered saline
SD: Standard deviation
