# Supplementary material for: Comparison of ELISA with electro-chemiluminescence technology for the qualitative and quantitative assessment of serological responses to vaccination
Source: Malar J. 2020 Apr 17;19:159. doi: 10.1186/s12936-020-03225-5 (PMC7165447; doi:10.1186/s12936-020-03225-5)
Supplement: Supplementary file 1 — Additional file 1: Figure S1. Overview of ECLIA assay platform. (Panel A) Experimental steps for assay setup. Biotinylated antigens are coupled with proprietary U-PLEX linkers in separate tubes. Once coupling is complete, U-PLEX-coupled antigens are combined into a cocktail and the assay plates coated. Each U-PLEX linker can only bind to its respective spot (color coded in Figure); up to 10 antigens can be coated per well (top view plate well). (Panel B) Overview of all steps to complete the assay: antigen-coated wells are incubated with diluted serum or plasma. Antigen-specific antibodies will bind to the antigen and the binding visualized by adding a Sulfo-Tag-labeled secondary antibody and substrate. (Panel C) Data acquisition. Plate is inserted into reader which will deliver an electric pulse that activates the substrate. A high resolution camera measures the luminescence above each spot. [file 12936_2020_3225_MOESM1_ESM.docx]

Additional file data Bolton et al.


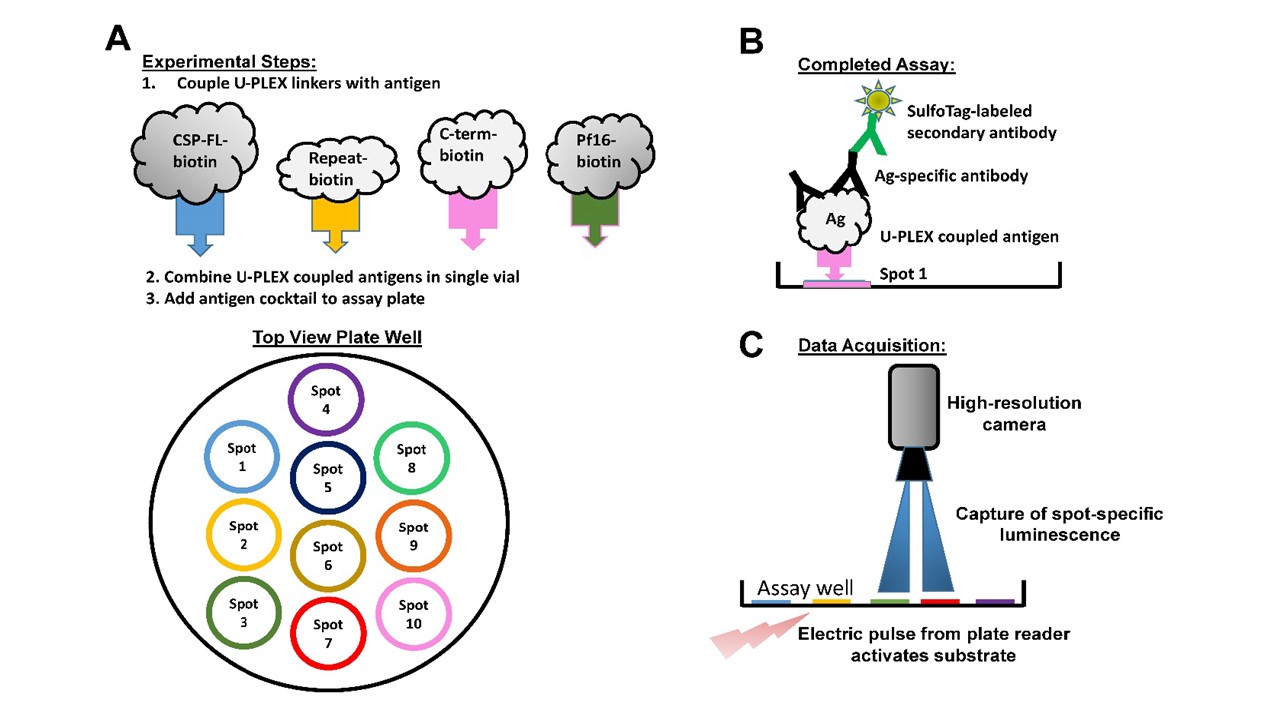


Additional file 1: Figure S1. Overview of ECLIA assay platform. (Panel A) Experimental steps for assay setup. Biotinylated antigens are coupled with proprietary U-PLEX linkers in separate tubes. Once coupling is complete, U-PLEX-coupled antigens are combined into a cocktail and the assay plates coated. Each U-PLEX linker can only bind to its respective spot (color coded in Figure); up to 10 antigens can be coated per well (top view plate well). (Panel B) Overview of all steps to complete the assay: antigen-coated wells are incubated with diluted serum or plasma. Antigen-specific antibodies will bind to the antigen and the binding visualized by adding a Sulfo-Tag-labeled secondary antibody and substrate. (Panel C) Data acquisition. Plate is inserted into reader which will deliver an electric pulse that activates the substrate. A high resolution camera measures the luminescence above each spot.
